# Influence of Different Multilayer Zirconia Materials on the In Vitro Load‐Bearing Capacity of Resin‐Bonded Fixed Partial Dentures in the Posterior Region

**DOI:** 10.1111/jerd.70191

**Published:** 2026-05-16

**Authors:** Moritz Waldecker, Maria Sophie Heller, Stefan Rues, Peter Rammelsberg, Wolfgang Bömicke

**Affiliations:** ^1^ Department of Prosthetic Dentistry Heidelberg University Hospital, University of Heidelberg Heidelberg Germany

**Keywords:** aging, load‐bearing capacity, multilayer zirconia, nesting position, resin‐bonded fixed partial dentures

## Abstract

**Objective:**

Comparison of the load‐bearing capacity and failure pattern of posterior resin‐bonded fixed partial dentures (RBFPDs) differing in retainer design, zirconia material, and vertical nesting position.

**Materials and Methods:**

RBFPDs replacing a maxillary first molar were fabricated with 2 retainer designs (inlay‐retained, IR; wing‐retained, WR) and of 2 multilayer zirconia materials (3Y‐TZP and 5Y‐PSZ, 3Y/5Y; 5Y‐PSZ, 5Y). For 3Y/5Y blanks, 2 vertical nesting positions (center; top) were evaluated. For each group, 16 monolithic RBFPDs were fabricated. Half of the RBFPDs underwent artificial aging. Load‐bearing capacity (F_u_) was tested under oblique loading. The data were statistically analyzed using Welch ANOVA and Dunnett‐T3 post hoc tests (*α* = 0.05).

**Results:**

No decementations or defects were observed after aging. Mean F_u_ ranged between 684 and 1216 N for IR‐RBFPDs and 1025–1617 N for WR‐RBFPDs. There was a clear hierarchy between the subgroups, with the lowest F_u_ recorded for restorations made of 5Y zirconia and the highest F_u_ for restorations fabricated from a centric position in 3Y/5Y blanks. Aging had no influence (*p* ≥ 0.526).

**Conclusions:**

Posterior RBFPDs made of multilayer 3Y‐TZP/5Y‐PSZ zirconia can be recommended for clinical trials. Vertical positioning in the blank influences the safety margin of the load‐bearing capacity. The use of 5Y‐PSZ should be critically scrutinized for this indication.

**Clinical Significance:**

Posterior resin‐bonded fixed partial dentures made of monolithic zirconia (3Y‐TZP) are a valuable treatment option. The opacity of the restorations limits their esthetics. Multilayer zirconia materials with color and/or material gradient can further improve the esthetics of monolithic zirconia restorations. Resin‐bonded fixed partial dentures made of multilayer zirconia materials differing in color and/or material gradient might be a viable alternative to restorations made of 3Y‐TZP zirconia in the posterior region.

## 1 Introduction

Improvements in prevention programs have led to a reduction of the experience of caries and the number of lost teeth in the population [1, 2]. As a result, the indication for minimally invasive treatment methods has increased. In the interrupted dental arch, a missing tooth today can be restored by various treatment alternatives that are considered prognostic equivalent: conventional fixed partial dentures (FPDs), implant‐supported single crowns, or resin‐bonded fixed partial dentures (RBFPDs) [3].

Survival rates of 94% after 5 years and 89% after 10 years are reported for the well‐studied conventional FPDs and implant‐supported single crowns [3]. For metal‐ceramic RBFPDs, the medium‐term survival rate has been reported ranging from 88% to 98% [4, 5, 6, 7]. Assuming proper (retentive) preparation, metal‐ceramic RBFPDs have been used with a 10‐year survival rate of between 83% and 95% [6, 8]. Debonding and chipping of the veneering ceramic have been found to be the most common technical complications of metal‐ceramic RBFPDs [4, 7]. Patients have generally rated both the function and the esthetics of metal‐ceramic RBFPDs as very good [6, 9]. However, it seems likely that patients would opt for a completely tooth‐colored restoration if it were available. Consequently, monolithic ceramics, such as lithium disilicate and zirconia, have been tested to improve esthetics by eliminating visible metal retainers and to improve technical robustness by avoiding veneers in load‐bearing areas, while maintaining low invasiveness. Inlay‐retained RBFPDs made of zirconia have been found to have a higher load‐bearing capacity than metal‐ceramic RBFPDs and ceramic RBFPDs made of lithium disilicate [10]. For RBFPDs made of 3 mol% yttria‐stabilized tetragonal zirconia polycrystal (3Y‐TZP), a mean load‐bearing capacity of over 1000 N was found for inlay‐retained RBFPDs (IR‐RBFPDs) [11, 12] and over 2000 N for wing‐retained RBFPDs (WR‐RBFPDs) [12] when tested for the restoration for the replacement of the first molar, which exceeds the limit value of 500 N required for the highly loaded posterior region [13, 14, 15].

Clinically, RBFPDs in the posterior region made of monolithic zirconia show promising short‐term survival rates of between 88.1% and 100% [16, 17, 18, 19, 20]. In these studies, in addition to the retainers, the pontics were monolithically designed [16, 17, 19, 20] or vestibulary veneered [18].

The optical properties of 3Y‐TZP are characterized by a high level of opacity. To improve the esthetic properties of zirconia, manufacturers have developed new zirconia materials with different proportions of yttria. In principle, the higher the proportion of yttria, the greater the translucency and the lower the flexural strength. Further optimization of the esthetic properties involved developing zirconia with a color (one zirconia with a specific proportion of yttria) or strength gradient (combination of different yttria‐doped zirconia materials), or both. However, there is currently no preclinical or clinical data on the suitability of such multilayer zirconia materials for the fabrication of RBFPDs in the posterior region.

Therefore, the purpose of this in vitro study was to compare the load‐bearing capacity and first damage of posterior monolithic IR‐RBFPDs and WR‐RBFPDs made of 2 different multilayer zirconia materials. The research hypotheses were that there would be significant differences in this regard (1) between the two RBFPD types and (2) between RBFPDs differing in zirconia material and/or vertical nesting position.

## 2 Material and Methods

IR‐RBFPDs and WR‐RBFPDs were tested in accordance with a pre‐established methodology. The methods described by Bömicke et al. were followed for the preparation of the abutment teeth, sample and test model production, aging process, and load‐bearing capacity test [12]. All necessary steps were carried out by a single operator (M.S.H.).

RBFPDs replaced a maxillary first molar of an anatomic study model (ANA‐4; Frasaco) with the second premolar and the second molar serving as abutment teeth.

**FIGURE 1:**
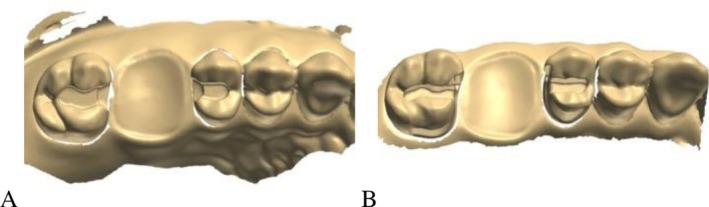
3D view of prepared teeth to accommodate a RBFPD with two different retainer designs. (A) Inlay. (B) Adhesive wing.

The abutment teeth for IR‐RBFPDs were prepared with a 2‐surface inlay cavity, following the guidelines for fabricating ceramic inlay‐supported prostheses [21]. Following the anatomy of the central fissure, an occlusal box was prepared with a depth of 1.5 mm measured from the floor to the central groove, and a width of 2.0 mm. The proximal box was prepared 2 mm deeper than the occlusal box, with an axial reduction of 1.5 mm and a width of 3 mm (Figure 1A).

The abutment teeth for WR‐RBFPDs were prepared with a chamfer finish line. Proximal, palatal, and occlusal abutment tooth substance was reduced by 0.5 mm. As described for posterior metal‐ceramic resin bonded fixed partial dentures, an occlusal box (depth, 1.5 mm; width, 1.5 mm) and a proximal groove (length, 4.0 mm) were prepared as retentive elements (Figure 1B).

In general, the axial walls were prepared using a paralleling device with a preparation angle of 4 degrees. The internal line angles were rounded and the preparations were finished using a fine‐grit (30 μm) diamond rotary instrument (Komet, Gebr. Brasseler). Abutment tooth reduction was evaluated using a silicone index (Flexitime Easy Putty, Heraeus Kulzer) and measured using a periodontal probe with a millimeter scale (Parodontometer PCPUNC15, Hu‐Friedy).

The RBFPD design process resulted in an identical external geometry of the pontic across all tested groups and identical external retainer geometries within corresponding subgroups. Therefore, IR‐RBFPDs and WR‐RBFPDs were first designed with a dental design software (Dental Designer; 3Shape) based on the original situation determined from a scan of the completely dentate study model with unprepared abutment teeth (ANA‐4; Frasaco). The connector cross‐sections were set at least 12 mm^2^. The connectors of the RBFPDs had the following height and width dimensions: IR‐RBFPD, mesial connector 4.4 × 1.6 mm and distal connector 5.1 × 4.2 mm. WR‐RBFPD, mesial connector 5.8 × 4.6 mm and distal connector 5.7 × 4.1 mm. For all RBFPDs, the pontic was provided with a standardized loading site (identical in shape and position) on the mesio‐palatal cusp of the pontic, which was tilted 60 degrees relative to the insertion direction (Geomagic Design X, software version: 2020; 3D Systems).

**FIGURE 2:**
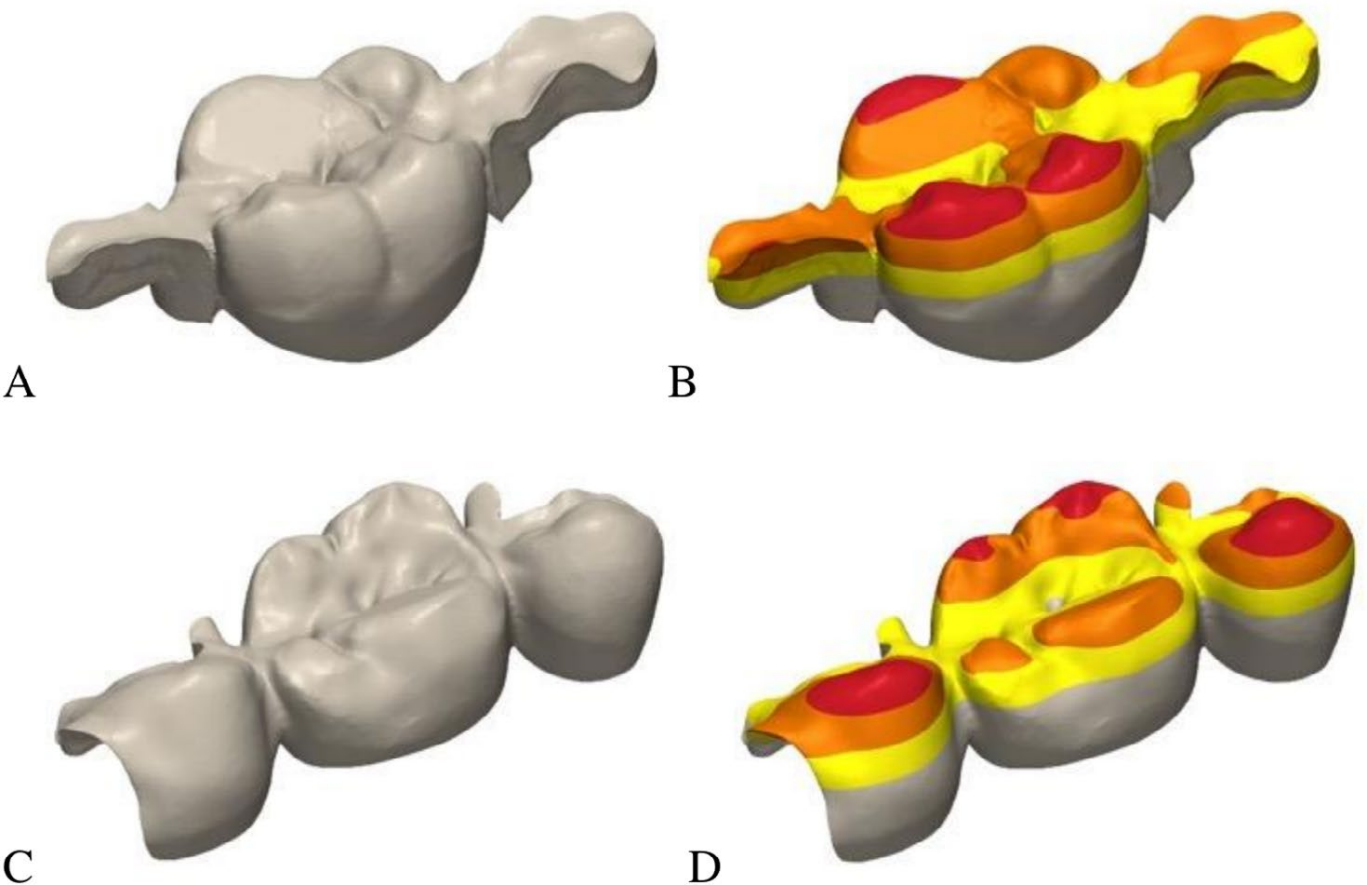
Nesting positions of RBFPDs in the 3Y/5Y blank. Red, 100% 5Y‐PSZ zirconia. Orange, 33% 3Y‐TZP and 67% 5Y‐PSZ. Yellow, 67% 3Y‐TZP and 33% 5Y‐PSZ. White, 100% 3Y‐TZP. (A) Inlay‐retained RBFPD, center position. (B) Inlay‐retained RBFPD, top position. (C) Wing‐retained RBFPD, center position. (D) Wing‐retained RBFPD, top position.

All RBFPDs were milled (Cercon brain expert; DeguDent) from blanks of different multilayer zirconia materials: a blank with color and material gradient combining 3Y‐TZP and 5 mol% yttria‐doped partially stabilized zirconia, 5Y‐PSZ (3Y/5Y; Cercon ht. ML; Dentsply Sirona) and a 5Y‐PSZ blank with color gradient (5Y; Cercon xt ML; Dentsply Sirona). For the 3Y/5Y blank, two different nesting positions were distinguished. RBFPDs were positioned either in the center or at the top of the blank (Figure 2A–D). For the 5Y blank, a vertical nesting position variation was not applicable. The 5Y blank consists of a single, homogeneous material. Therefore, changing the vertical nesting position does not affect the material composition. After milling, RBFPDs were sintered at 1500°C (Cercon heat plus P8; DeguDent) and glazed in accordance with the manufacturers' instructions (Universal Overglaze with Universal Stain and Glaze; both Dentsply Sirona).

The prepared abutment teeth were fitted with standardized roots (length, 10 mm; cervical/apical cross‐sectional areas, 6 × 10 mm to 3 × 5 mm) and duplicated in cobalt‐chromium (CoCr) alloy (Biosil L; DeguDent). To simulate tooth mobility, the standardized roots of the abutment teeth were fitted with heat‐shrink tubing (Protect; Bahag) and sealed apically with 2 mm low‐viscosity polyvinyl siloxane impression material (Flexitime Correct Flow; Heraeus Kulzer). To finalize the test model, the CoCr abutment teeth were embedded with the respective RBFPD fixed to a parallelometer as a positioning aid in an aluminum block using an autopolymerizing resin (Technovit 4071; Heraeus Kulzer).

Before definitive cementation, metal abutment teeth were airborne‐particle abraded using 50 μm alumina particles at pressures of 0.3 MPa; remnants of the abrasive material were removed using oil‐free air.

Aluminum blocks with metal abutment teeth were heated to 47°C in an incubator (Heraeus Function Line; Heraeus Kulzer) to maintain a temperature of approximately 37°C during cementation.

The RBFPD bonding surfaces were conditioned in accordance with an already established procedure [22], including coating with silica (Rocatec system; 3 M ESPE) and applying a universal primer (Clearfil Ceramic Primer; Kuraray Noritake Dental) for 60 s. All RBFPDs were cemented under a constant axial load of 200 N (Z005; ZwickRoell) with autopolymerizing 10‐methacryloyloxydecyl dihydrogen phosphate (MDP)‐based resin cement (Panavia 21; Kuraray Noritake Dental). After removing the excess cement, the restoration margins were covered with polymerization‐activating gel (Oxyguard; Kuraray Noritake Dental). After 4 min, the load was removed, and the test specimens were stored for additional 6 min at 37°C in the incubator to allow complete polymerization of the cement. Following adhesive cementation, all RBFPDs were stored for 24 ± 1 h at 37°C and 100% humidity. Half of the RBFPDs per group underwent artificial aging consisting of 30 days of storage in deionized water at 37°C including 10,000 thermocycles (Thermocycler TC 1; SD Mechatronik), with bath temperatures of 6.5°C and 60°C and a respective dwell time of 45 s, as well as 1,200,000 cycles of mechanical loading (mastication simulator CS4 with additional spring‐damper system; SD Mechatronik, purely vertical movement with mass *m* = 9 kg, descending speed v = 30 mm/s; spring *k* = 43 N/mm; damper *d* = 135 Ns/m; leading to a force magnitude of magnitude F_max_ = 108 N). The loaded cusp was oriented horizontally during mechanical loading, and a steel sphere with a diameter of 6 mm was used to apply a load perpendicular to the pontic's flattened mesiopalatal plane 2 mm below the cusp. The result was a standardized loading vector tilted by 30° in relation to the tooth axis [23, 24].

The load‐bearing capacity (F_u_) of each RBFPD was determined using a universal testing machine (Z005; ZwickRoell; cross‐head speed of 0.5 mm/min). The load application to the pontic was identical to that during mastication simulation. Software (testXpert II; Zwick) was used to determine test force at prosthesis failure (F_u_: load‐bearing capacity), defined as the maximum test force recorded before the test force dropped to below 30% of the maximum force. During testing, RBFPDs were visually observed for structural changes, and prosthesis structure‐borne sound signals were captured with a contact microphone to detect initial damage events. Finally, fracture patterns of failed RBFPDs were qualitatively documented with a digital microscope (eScope DP‐M07; Oriental Inspiration Limited).

F_u_ was recorded as mean, standard deviation (SD), minima (Min), medians, and maxima (Max).

As part of a power analysis, a sample size determination for a two samples *t*‐test was performed. The calculation was based on fracture load data reported by Bömicke et al. [12] and on supplementary biaxial flexural strength data obtained with the materials investigated in the present study: 761 ± 89 MPa for monolithic 5Y‐PSZ or enamel layer of 3Y/5Y zirconia blanks, 886 ± 117 MPa for the upper part of the transition layer within 3Y/5Y zirconia blanks, and 1431 ± 181 MPa for monolithic 3Y‐TZP or dentin layer within 3Y/5Y zirconia blanks. Power was set at 80% and the level of significance at *α* = 0.05. Bömicke et al. reported mean fracture loads of 1215 ± 244 N for IR‐RBFPDs and 2191 ± 381 N for WR‐RBFPDs. For RBFPDs made of 3Y‐TZP in this study, the same mean values and standard deviation should be observed. By the rule of three, mean fracture forces for RBFPDs made of 5Y‐PSZ or 3Y/5Y multilayer zirconia were estimated depending on the lowest material strength present in the critical RBFPD regions in contrast to the strength of 3Y‐TZP. The resulting minimum sample size was *n* = 6 for IR‐RBFPDs and *n* = 5 for WR‐RBFPDs.

It should be acknowledged that the power analysis was conservatively based on a two‐sample comparison. However, in the final analysis (Welch ANOVA with Dunnet‐T3 tests), several groups were compared simultaneously. To account for this discrepancy, as well as for the variability inherent to in vitro testing and possible deviations from normal distribution assumptions, the final sample size was increased to *n* = 8 specimens per group.

Data was evaluated by use of statistical software (IBM SPSS Statistics 28; IBM). The data were checked for normal distribution using the Shapiro–Wilk test and by analyzing the Q‐Q plots. The Levene test was used to check for equality of variances among the test groups. Because the data exhibited a normal distribution with simultaneous variance inequality, Welch ANOVA and Dunnett‐T3 tests for pairwise group comparisons were used. The significance level was *p* = 0.05.

## 3 Results

**FIGURE 3:**
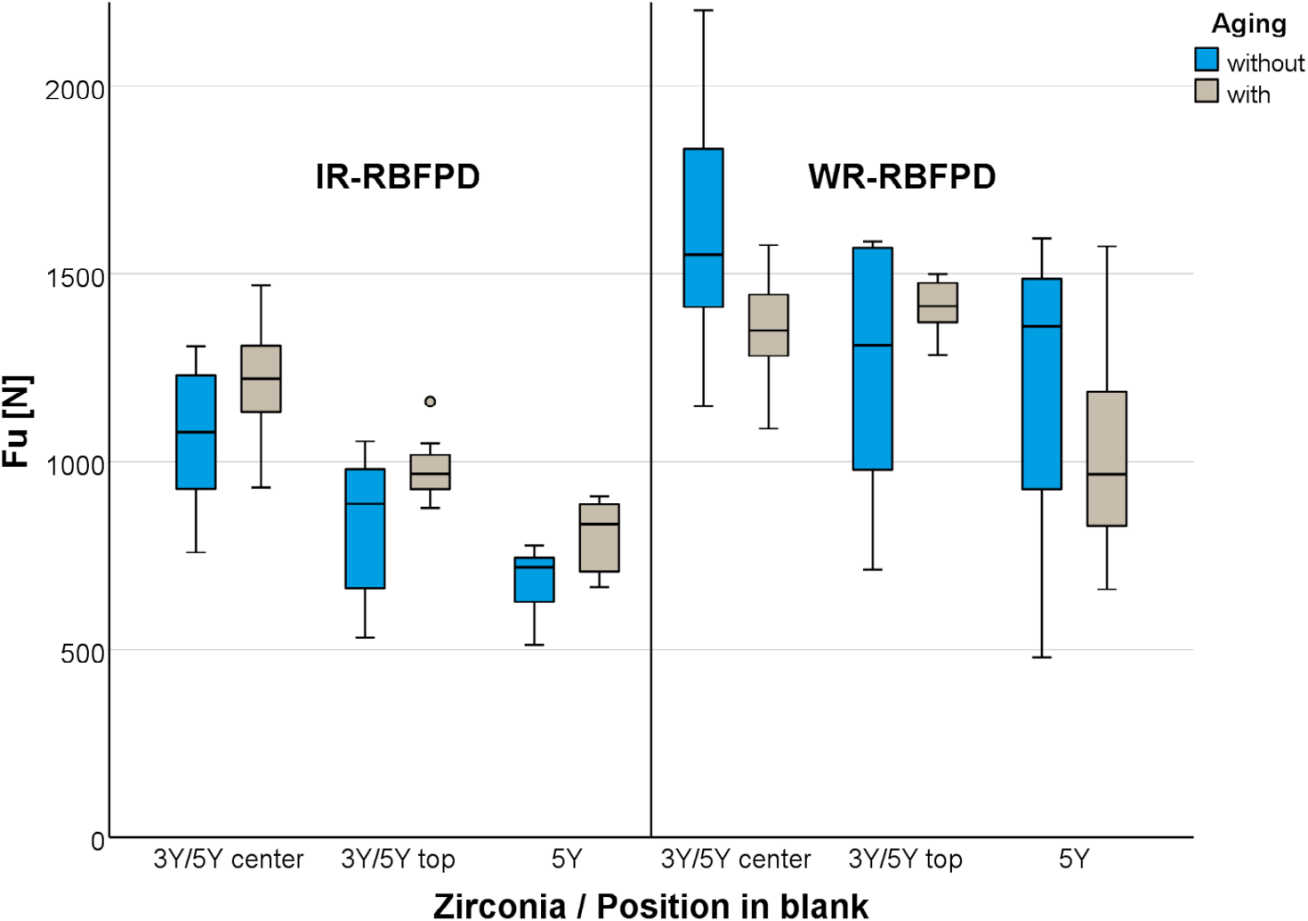
Box and whisker plots for RBFPDs with different retainer designs at definitive failure (F_u_).

Test forces at failure (F_u_) for RBFPDs are given in Figure 3, and Table 1. No decementations, cracks, or fractures of the RBFPDs were observed after artificial aging. During load‐bearing capacity testing, no initial damage was detected in any of the restorations. All RBFPDs failed definitively. The comparison of the individual subgroups descriptively shows that WR‐RBFPDs had a higher load‐bearing capacity than IR‐RBFPDs. For each type of restoration, there was a clear hierarchy between the subgroups, with the lowest F_u_ recorded for restorations made of 5Y zirconia and the highest F_u_ given for RBFPDs fabricated from a centric position in 3Y/5Y blanks. Restorations subjected to artificial aging tended to have a higher load‐bearing capacity.

**TABLE 1:** Test forces at failure (F_u_) for all RBFPDs.

Retainer design	Zirconia	Position in blank	Aging	F_u_ [*N*]					
				Mean (±SD)	Min	Median	Max	95% CI	
								LCL	UCL
Inlay	3Y/5Y	Center	Without	1067 (±192)	759	1078	1307	907	1228
			With	1216 (±163)	932	1222	1469	1080	1352
		Top	Without	831 (±191)	533	888	1054	672	991
			With	983 (±87)	877	968	1160	910	1056
	5Y		Without	684 (±88)	513	718	777	610	758
			With	804 (±96)	667	834	908	723	885
Wing	3Y/5Y	Center	Without	1617 (±332)	1148	1551	2202	1340	1895
			With	1352 (±150)	1088	1349	1576	1227	1477
		Top	Without	1251 (±341)	713	1309	1586	966	1537
			With	1413 (±73)	1285	1414	1499	1352	1474
	5Y		Without	1203 (±392)	479	1360	1594	875	1531
			With	1025 (±288)	660	967	1573	785	1266

Abbreviations: 95% CI, 95% confidence interval; F_u_, fracture load; LCL, lower confidence level; Max, maximum; Min, minimum; SD, standard deviation; UCL, upper confidence level.

Welch ANOVA revealed a significant difference between groups (*p* ≤ 0.001). Group comparison only showed differences for certain comparisons when considering the factors retainer design and material. Concerning the factor retainer design, a significant difference between IR‐RBFPDs and WR‐RBFPDs fabricated of 3Y/5Y with top position in the blank (*p* < 0.001) was observed. Concerning the factor material, significant differences were only determined for IR‐RBFPDs. IR‐RBFPDs made of 3Y/5Y with center position in the blank exhibited a significantly higher load‐bearing capacity than IR‐RBFPDs made of 5Y (*p* ≤ 0.019). Aging had no statistically significant influence (*p ≥ 0*.526). All pairwise comparisons can be found in Table 2.

**TABLE 2:** Statistical significance of group differences and effect sizes.

		Mean differences	SE	*p*	Hegdes'g	95% CI	
						LCL	UCL
IR, 3Y/5Y center, wa	IR, 3Y/5Y top, wa	233	65	0.156	1.52	−50	516
	IR, 5Y center, wa	412	67	0.003	2.59	126	697
	WR, 3Y/5Y center, wa	−136	78	0.963	−0.68	−457	184
	WR, 3Y/5Y top, wa	−197	63	0.294	−1.37	−478	83
	WR, 5Y center, wa	190	117	0.977	0.58	−312	693
	IR, 3Y/5Y center, woa	148	89	0.976	0.63	−218	515
	IR, 3Y/5Y top, woa	384	89	0.034	1.63	19	749
	IR, 5Y center, woa	532	65	0.000	3.47	249	815
	WR, 3Y/5Y center, woa	−402	131	0.312	−1.08	−976	173
	WR, 3Y/5Y top, woa	−36	134	1.000	−0.09	−625	553
	WR, 5Y center, woa	13	150	1.000	0.03	−661	686
IR, 3Y/5Y top, wa	IR, 5Y center, wa	179	46	0.072	−1.26	−10	368
	WR, 3Y/5Y center, wa	−369	61	0.004	1.48	−631	−106
	WR, 3Y/5Y top, wa	−430	40	0.000	−2.15	−596	−264
	WR, 5Y center, wa	−42	106	1.000	−4.26	−536	452
	IR, 3Y/5Y center, woa	−84	75	1.000	−0.14	−415	247
	IR, 3Y/5Y top, woa	152	74	0.843	−0.39	−177	481
	IR, 5Y center, woa	299	44	0.000	0.72	119	480
	WR, 3Y/5Y center, woa	−634	122	0.028	2.62	−1206	−63
	WR, 3Y/5Y top, woa	−268	125	0.782	−1.77	−855	319
	WR, 5Y center, woa	−220	142	0.978	−0.73	−895	455
IR, 5Y center, wa	WR, 3Y/5Y center, wa	−548	63	0.000	−2.21	−814	−282
	WR, 3Y/5Y top, wa	−609	43	0.000	−1.53	−786	−432
	WR, 5Y center, wa	−221	107	0.828	−3.15	−715	272
	IR, 3Y/5Y center, woa	−263	76	0.183	−5.78	−596	69
	IR, 3Y/5Y top, woa	−27	76	1.000	−0.71	−358	303
	IR, 5Y center, woa	120	46	0.526	−1.22	−69	310
	WR, 3Y/5Y center, woa	−813	122	0.006	−0.13	−1384	−243
	WR, 3Y/5Y top, woa	−447	125	0.190	1.02	−1034	139
	WR, 5Y center, woa	−399	143	0.456	−2.27	−1074	275
WR, 3Y/5Y center, wa	WR, 3Y/5Y top, wa	−61	59	1.000	−0.45	−320	197
	WR, 5Y center, wa	327	115	0.407	1.01	−172	826
	IR, 3Y/5Y center, woa	285	86	0.200	1.23	−72	641
	IR, 3Y/5Y top, woa	521	86	0.002	2.26	166	876
	IR, 5Y center, woa	668	61	0.000	4.58	405	931
	WR, 3Y/5Y center, woa	−266	129	0.835	−0.72	−838	307
	WR, 3Y/5Y top, woa	101	132	1.000	0.27	−487	688
	WR, 5Y center, woa	149	148	1.000	0.35	−524	822
WR, 3Y/5Y top, wa	WR, 5Y center, wa	388	105	0.166	1.25	−107	883
	IR, 3Y/5Y center, woa	346	73	0.038	1.64	16	675
	IR, 3Y/5Y top, woa	582	72	0.001	2.78	254	909
	IR, 5Y center, woa	730	41	0.000	6.74	562	897
	WR, 3Y/5Y center, woa	−204	120	0.951	−0.57	−777	368
	WR, 3Y/5Y top, woa	162	123	0.996	0.44	−426	750
	WR, 5Y center, woa	210	141	0.984	0.5	−466	886
WR, 5Y center, wa	IR, 3Y/5Y center, woa	−42	122	1.000	−0.14	−557	473
	IR, 3Y/5Y top, woa	194	122	0.984	0.66	−320	708
	IR, 5Y center, woa	342	106	0.285	1.46	−152	835
	WR, 3Y/5Y center, woa	−592	155	0.084	−1.44	−1231	47
	WR, 3Y/5Y top, woa	−226	158	0.996	−0.54	−876	424
	WR, 5Y center, woa	−178	172	1.000	−0.38	−894	538
IR, 3Y/5Y center, woa	IR, 3Y/5Y top, woa	236	96	0.613	0.95	−156	628
	IR, 5Y center, woa	384	75	0.019	2.24	53	715
	WR, 3Y/5Y center, woa	−550	136	0.072	−1.45	−1132	32
	WR, 3Y/5Y top, woa	−184	138	0.998	−0.47	−780	412
	WR, 5Y center, woa	−136	154	1.000	−0.31	−813	542
IR, 3Y/5Y top, woa	IR, 5Y center, woa	148	74	0.869	0.87	−181	477
	WR, 3Y/5Y center, woa	−786	136	0.005	−2.07	−1368	−204
	WR, 3Y/5Y top, woa	−420	138	0.318	−1.08	−1016	176
	WR, 5Y center, woa	−372	154	0.647	−0.85	−1049	306
IR, 5Y center, woa	WR, 3Y/5Y center, woa	−934	122	0.002	−2.61	−1505	−363
	WR, 3Y/5Y top, woa	−568	125	0.060	−1.55	−1155	19
	WR, 5Y center, woa	−519	142	0.178	−1.24	−1195	156
WR, 3Y/5Y center, woa	WR, 3Y/5Y top, woa	366	168	0.792	0.84	−324	1056
	WR, 5Y center, woa	414	182	0.729	0.86	−334	1163
WR, 3Y/5Y top, woa	WR, 5Y center, woa	48	184	1.000	0.1	−707	804

Abbreviations: 95% CI, 95% confidence interval; IR, Inlay retained; LCL, lower confidence level; SE, standard error; UCL, upper confidence level; wa, with aging; woa, without aging; WR, wing retained.

**FIGURE 4:**
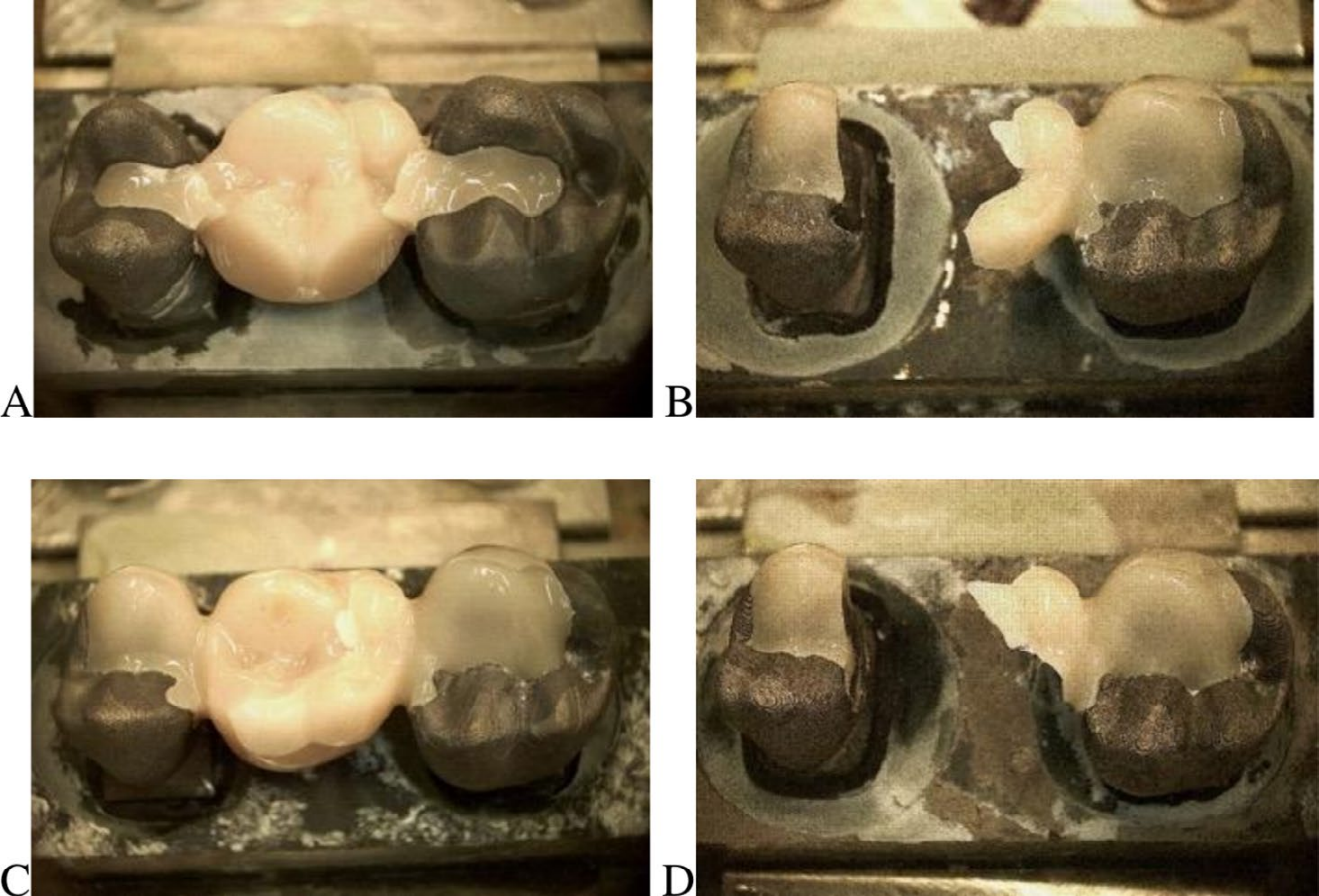
Typical fracture patterns for RBFPDs with different retainer designs. (A) Inlay‐retained RBFPD. (B–D) Wing‐retained RBFPD.

All RBFPDs failed cohesively, with complete fracture in the distal and/or mesial connector region. Figure 4 shows examples of typical fracture patterns.

## 4 Discussion

This study evaluated the load‐bearing capacity of IR‐ and WR‐RBFPDs made from different zirconia materials, considering the position of the restoration in the blank as a variable for multilayer zirconia with a strength gradient. Because Welch ANOVA revealed significant differences among test groups, the null hypothesis was rejected. The minimum mean fracture load was 684 N for IR‐RBFPDs and 1025 N for WR‐RBFPDs. These values exceed the maximum occlusal force on a single tooth in the molar region (approximately 500 N) [13, 14, 15]. Therefore, complete fracture appears to be an unlikely cause of clinical failure for these types of restorations. However, fabrication of RBFPDs from 5Y‐PSZ blanks should be critically evaluated, as the safety margin is small here, especially with IR‐RBFPDs.

In the present study, there was no group made from homogenous 3Y‐TZP blank, since the dentin layer of the used multilayer blank (Cercon ht. ML; Dentsply Sirona) consists of the same 3Y‐TZP material of the manufacturer. With a centric nesting position, the entire restoration is milled from the dentin layer, as can be seen in Figure 2A,C. Thus, these restorations can be compared to those milled from a 3Y‐TZP blank. Bömicke et al. reported mean fracture loads of 1215 N for IR‐RBFPDs and 2191 N for WR‐RBFPDs. Both had been fabricated from 3Y‐TZP (Cercon ht.; Dentsply Sirona) and had been artificially aged. Compared to the RBFPDs fabricated from multilayer blanks in a centric position, there is good agreement for the IR‐RBFPDs (1216 N). In contrast to the study by Bömicke et al., the mean fracture load for WR‐RBFPDs is lower, at 1352 N (with aging) and 1617 N (without aging). The reason for the discrepancy between the two studies is difficult to determine. The differences may be due to variations in the abutment teeth's manufacturing process or to a different manufacturer of the sample.

RBFPDS subjected to artificial aging tended to have a slightly higher load‐bearing capacity compared to restorations without aging. It could be speculated that the test set‐up adapted to the applied load to a small degree during chewing simulation. Furthermore, t/m transformation at the surface of a zirconia restoration can cause a higher fracture load due to compressive stress formation [25].

The abutment teeth in the experimental model were fabricated from CoCr alloy and were resiliently supported. Metal abutment teeth were selected to ensure standardization, thereby minimizing variability associated with natural teeth, and because natural teeth would likely fail prior to the restorations under the oblique loading conditions employed in this study. The combination of abutment teeth support and abutment teeth material properties also influences the occurrence, localization, and magnitude of tensile stresses [11]. Relative to the reference condition (natural tooth dentin with tooth mobility), abutment teeth composed of resin (−50% to −57%) or composite resin (−11% to −23%) in conjunction with resilient support led to an underestimation of the relative fracture resistance for IR‐RBFPDs [11]. In contrast, the utilization of CoCr abutment teeth resulted in only slight under‐ or overestimations, ranging from −6% to +15% [11]. In comparison for rigidly supported abutment teeth, an overestimation of 30%–45% of the relative fracture resistance has been reported when employing natural dentin, and an overestimation by a factor of 1.5–1.95 when using CoCr abutment teeth [11].

In this study, the specimens were subjected to oblique and eccentric loading to simulate the most critical loading conditions. When IR‐RBFPDs were loaded eccentrically, a reduction in failure load from 1749 N (centric loading) to 880 N (eccentric loading) has been documented [23]. A finite element analysis comparing axial and oblique load application for monolithic and veneered IR‐RBFPDs revealed no significant difference between the two loading conditions for resiliently supported abutment teeth. However, if the abutment teeth were supported rigidly, the fracture load of both the framework (zirconia, up to 130%) and the veneering ceramic (up to 300%) was overestimated. The greater the elastic modulus of the abutment tooth material, the more pronounced the overestimation of the fracture load [11].

All RBFPDs in the present study fractured in the connector area. Previous studies on monolithic ceramic IR‐RBFPDs made of zirconia have shown that the highest tensile stresses occur on the bottom side of the connectors, and that crack initiation begins here [11, 24].

**FIGURE 5:**
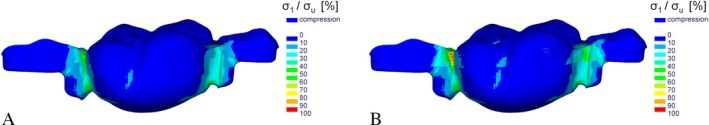
First principal stresses related to flexural strength for maximum load application to an inlay‐retained RBFPD. (A) Homogeneous restorations (3Y/5Y with center position or 5Y). (B) Inhomogeneous restorations (3Y/5Y with top position).

A clear hierarchy was found between the material subgroups for each type of restoration, with the lowest fracture loads occurring in restorations fabricated from 5Y‐PSZ and the highest in RBFPDs made from a centered position in 3Y/5Y zirconia blanks (entire RBFPD in the 3Y‐TZP section). The gradation of the fracture loads between the subgroups suggests that there is an influence of the material. To facilitate discussion of the study results, a calculation was performed for IR‐FPDs based on an FE analysis previously published by Waldecker et al. [11] It considered a homogeneous restoration (3Y/5Y in the center position or 5Y) and an inhomogeneous restoration (3Y/5Y in the top position). In the homogeneous restoration, consisting either solely of 3Y‐TZP or 5Y‐PSZ, critical tensile stresses reaching the material's strength occur only on the bottom side of the connectors (Figure 5A). In the case of inhomogeneous zirconia restorations showing lower flexural strength in the upper part compared to the lower part, critical tensile stresses occur on both the bottom and the top side (Figure 5B). For the illustration in Figure 5B a simplification was made by splitting the restoration only in 2 material zones, and correlating the transition layers with the weaker 5Y‐PSZ. The manufacturer specifies the following flexural strength values for the different zirconia types: 5Y zirconia = 750 MPa and 3Y/5Y zirconia = 750–1200 MPa. Again, for better interpretation of the fracture load tests and the FE analysis, additional biaxial tests were performed with the materials used in this study. The following flexural strength values were determined: 5Y zirconia = 761 ± 89 MPa, 3Y/5Y zirconia (1/3 3Y‐TZP / 2/3 5Y‐PSZ) = 886 ± 117 MPa, and 3Y/5Y zirconia (whole restoration in the 3Y‐TZP area) = 1431 ± 181 MPa. The results from FE analysis in combination with the determined flexural strength values show that in inhomogeneous restorations, critical tensile stresses also occur on the upper side and thus in the area of the ceramic with lower flexural strength. The failure thus initiates at the upper side of the connector. Consequently, as seen in the fracture load tests, the restorations made of 3Y/5Y with top position will fail at lower fracture loads than restorations made of 3Y/5Y with center position. It should be noted at this point that the results of the finite element analysis apply specifically to IR‐RBFPDs. It is not feasible to extrapolate quantitative stress values based on these results to the WR‐RBFPD design due to the differing geometry in the connector region, which is caused by the characteristic inlay or adhesive wing geometry. Since the highest tensile stresses typically occur in the connector region, geometric differences are particularly critical here and can have a significant impact on the fracture load of RBFPDs.

High variability was observed in some groups, particularly in WR‐RBFPDs fabricated from 3Y/5Y zirconia with top position after aging and in WR‐RBFPDs made of 5Y zirconia with and without aging. The coefficient of variation (CV) ranged between 27% and 33%. The elevated CV can be attributed to material‐related characteristics in combination with minimal inaccuracies in fit. Due to its increased cubic phase content, 5Y zirconia exhibits reduced transformation toughening and consequently lower intrinsic strength compared to conventional 3Y‐TZP. This inherent material weakness increases its susceptibility to stress concentrations. As a result, even minor deviations in internal or marginal fit, slight variations in cement space, or minimal discrepancies in adaptation can lead to localized stress peaks that substantially affect fracture behavior. When combined with the comparatively lower mechanical strength of 5Y zirconia, such small fitting inaccuracies can lead to stress distribution differences within the restoration, thereby contributing significantly to the observed variability in fracture load values. The dispersion of results should therefore not be interpreted merely as random variation, but rather as an expression of the material's higher sensitivity to fit‐related deviations. Accordingly, the pronounced variability indicates reduced mechanical consistency within these groups and suggests limited predictability under functional loading conditions. The findings for these groups should therefore be interpreted critically.

In vitro studies are conducted under ideal test conditions that allow precise control of variables such as load application, loading direction, and tooth position. However, the controlled nature of the laboratory setting limits the transferability of the results to clinical situations. In clinical practice, the forces acting on RBFPDs are variable and can occur in different magnitudes and directions in contrast to the few critical loading conditions chosen for the in vitro tests. Therefore, it remains unclear to what extent the results obtained in vitro will influence the clinical performance of the tested RBFPDs. Furthermore, the transfer of the presented data to situations using different adhesive systems or replacing other teeth should be done with care. Future studies should therefore focus on the clinical testing of RBFPDs made of different multilayer zirconia materials.

## 5 Conclusions

Within the limitations of this current study, it was concluded that:

1. RBFPDs fabricated from multilayer zirconia materials demonstrated clinically acceptable load‐bearing capacity and are therefore suitable for clinical trials.
2. The use of 5Y‐PSZ as a framework material for RBFPDs requires critical evaluation, as the obtained failure load values were close to the forces associated with clinical molar occlusal loads.

## Conflicts of Interest

The authors declare no conflicts of interest.

## Data Availability

The data that support the findings of this study are available from the corresponding author upon reasonable request.

## References

[1] A. R. Jordan , H. Meyer‐Lueckel , K. Kuhr , D. Sasunna , K. Bekes , and U. Schiffner , “Caries Experience and Care in Germany: Results of the 6th German Oral Health Study (DMS * 6),” Quintessence International (Berlin) 56 (2025): S30–S39. 10.3290/j.qi.b5986212 40091720

[2] B. Wöstmann , S. Samietz , A. R. Jordan , K. Kuhr , I. Nitschke , and H. Stark , “Tooth Loss and Denture Status: Results of the 6th German Oral Health Study (DMS * 6),” Quintessence International (Berlin) 56 (2025): S60–S68. 10.3290/j.qi.b5986257 40091723

[3] B. E. Pjetursson , U. Bragger , N. P. Lang , and M. Zwahlen , “Comparison of Survival and Complication Rates of Tooth‐Supported Fixed Dental Prostheses (FDPs) and Implant‐Supported FDPs and Single Crowns (SCs),” Clinical Oral Implants Research 18, no. Suppl 3 (2007): 97–113. 17594374 10.1111/j.1600-0501.2007.01439.x

[4] D. S. Thoma , I. Sailer , A. Ioannidis , M. Zwahlen , N. Makarov , and B. E. Pjetursson , “A Systematic Review of the Survival and Complication Rates of Resin‐Bonded Fixed Dental Prostheses After a Mean Observation Period of at Least 5 Years,” Clinical Oral Implants Research 28 (2017): 1421–1432. 28191679 10.1111/clr.13007

[5] I. A. Alraheam , C. N. Ngoc , C. A. Wiesen , and T. E. Donovan , “Five‐Year Success Rate of Resin‐Bonded Fixed Partial Dentures: A Systematic Review,” Journal of Esthetic and Restorative Dentistry 31 (2019): 40–50. 30302909 10.1111/jerd.12431

[6] R. Handermann , P. Rammelsberg , and W. Bömicke , “Metal‐Ceramic Resin‐Bonded Fixed Partial Dentures After a Mean Observation Time of 7.5 Years,” International Journal of Prosthodontics 37 (2024): 157–165. 38648164 10.11607/ijp.8296

[7] B. E. Pjetursson , W. C. Tan , K. Tan , U. Bragger , M. Zwahlen , and N. P. Lang , “A Systematic Review of the Survival and Complication Rates of Resin‐Bonded Bridges After an Observation Period of at Least 5 Years,” Clinical Oral Implants Research 19 (2008): 131–141. 18070120 10.1111/j.1600-0501.2007.01527.x

[8] M. Behr , A. Leibrock , W. Stich , P. Rammelsberg , M. Rosentritt , and G. Handel , “Adhesive‐Fixed Partial Dentures in Anterior and Posterior Areas. Results of an On‐Going Prospective Study Begun in 1985,” Clinical Oral Investigations 2 (1998): 31–35. 9667152 10.1007/s007840050040

[9] N. H. Creugers and R. J. De Kanter , “Patients' Satisfaction in Two Long‐Term Clinical Studies on Resin‐Bonded Bridges,” Journal of Oral Rehabilitation 27 (2000): 602–607. 10931253 10.1046/j.1365-2842.2000.00553.x

[10] M. A. Kilicarslan , P. S. Kedici , H. C. Kucukesmen , and B. C. Uludag , “In Vitro Fracture Resistance of Posterior Metal‐Ceramic and All‐Ceramic Inlay‐Retained Resin‐Bonded Fixed Partial Dentures,” Journal of Prosthetic Dentistry 92 (2004): 365–370. 15507910 10.1016/j.prosdent.2004.07.001

[11] M. Waldecker , S. Rues , P. Rammelsberg , and W. Bömicke , “Validation of In‐Vitro Tests of Zirconia‐Ceramic Inlay‐Retained Fixed Partial Dentures: A Finite Element Analysis,” Dental Materials 35 (2019): e53–e62. 30686709 10.1016/j.dental.2019.01.017

[12] W. Bömicke , M. Waldecker , J. Krisam , P. Rammelsberg , and S. Rues , “In Vitro Comparison of the Load‐Bearing Capacity of Ceramic and Metal‐Ceramic Resin‐Bonded Fixed Dental Prostheses in the Posterior Region,” Journal of Prosthetic Dentistry 119 (2018): 89–96. 28533015 10.1016/j.prosdent.2017.03.006

[13] V. F. Ferrario , C. Sforza , G. Serrao , C. Dellavia , and G. M. Tartaglia , “Single Tooth Bite Forces in Healthy Young Adults,” Journal of Oral Rehabilitation 31 (2004): 18–22. 15125591 10.1046/j.0305-182x.2003.01179.x

[14] F. A. Fontijn‐Tekamp , A. P. Slagter , A. Van Der Bilt , et al., “Biting and Chewing in Overdentures, Full Dentures, and Natural Dentitions,” Journal of Dental Research 79 (2000): 1519–1524. 11005738 10.1177/00220345000790071501

[15] S. C. Regalo , C. M. Santos , M. Vitti , et al., “Evaluation of Molar and Incisor Bite Force in Indigenous Compared With White Population in Brazil,” Archives of Oral Biology 53 (2008): 282–286. 18031710 10.1016/j.archoralbio.2007.10.003

[16] W. Bömicke , F. Rathmann , P. Rammelsberg , and A. Zenthöfer , “Three‐Year Performance of Inlay‐Retained or Wing‐Retained Zirconia Resin‐Bonded Fixed Partial Dentures ‐ Results From a Randomized Clinical Pilot Study,” Journal of Dentistry 105807 (2025). 10.1016/j.jdent.2025.105807 40339895

[17] W. Bömicke , F. Rathmann , M. Pilz , et al., “Clinical Performance of Posterior Inlay‐Retained and Wing‐Retained Monolithic Zirconia Resin‐Bonded Fixed Partial Dentures: Stage One Results of a Randomized Controlled Trial,” Journal of Prosthodontics 30 (2021): 384–393. 32924240 10.1111/jopr.13258

[18] C. Yazigi and M. Kern , “Clinical Evaluation of Zirconia Cantilevered Single‐Retainer Resin‐Bonded Fixed Dental Prostheses Replacing Missing Canines and Posterior Teeth,” Journal of Dentistry 116 (2022): 103907. 34838845 10.1016/j.jdent.2021.103907

[19] A. T. Kasem , J. P. M. Tribst , M. Abo‐Madina , and W. Al‐Zordk , “Evaluation of Different Designs for Posterior Cantilever Zirconia Inlay‐Retained Fixed Dental Prostheses in Missing Tooth Replacement: Stage One Results With 18‐Month Follow‐Up Assessment,” Journal of Dentistry 137 (2023): 104688. 37669722 10.1016/j.jdent.2023.104688

[20] W. Y. H. Lam , T. W. Lim , M. J. Yu Yon , et al., “Posterior Two‐Unit Cantilevered Zirconia Resin‐Bonded Fixed Partial Dentures: A 3‐Year Prospective Single‐Arm Clinical Trial,” Journal of Dentistry 147 (2024): 105140. 38901823 10.1016/j.jdent.2024.105140

[21] M. C. Thompson , K. M. Thompson , and M. Swain , “The All‐Ceramic, Inlay Supported Fixed Partial Denture. Part 1. Ceramic Inlay Preparation Design: A Literature Review,” Australian Dental Journal 55 (2010): 120–127. 20604751 10.1111/j.1834-7819.2010.01214.x

[22] W. Bömicke , A. Schürz , J. Krisam , P. Rammelsberg , and S. Rues , “Durability of Resin‐Zirconia Bonds Produced Using Methods Available in Dental Practice,” Journal of Adhesive Dentistry 18 (2016): 17–27. 26814317 10.3290/j.jad.a35517

[23] C. Mehl , K. Ludwig , M. Steiner , and M. Kern , “Fracture Strength of Prefabricated All‐Ceramic Posterior Inlay‐Retained Fixed Dental Prostheses,” Dental Materials 26 (2010): 67–75. 19836829 10.1016/j.dental.2009.07.013

[24] Z. Zhang , M. Thompson , C. Field , W. Li , Q. Li , and M. V. Swain , “Fracture Behavior of Inlay and Onlay Fixed Partial Dentures—An In‐Vitro Experimental and XFEM Modeling Study,” Journal of the Mechanical Behavior of Biomedical Materials 59 (2016): 279–290. 26894661 10.1016/j.jmbbm.2016.01.035

[25] M. Okada , H. Taketa , Y. Torii , M. Irie , and T. Matsumoto , “Optimal Sandblasting Conditions for Conventional‐Type Yttria‐Stabilized Tetragonal Zirconia Polycrystals,” Dental Materials 35, no. 1 (2019): 169–175. 30502226 10.1016/j.dental.2018.11.009

